# First report of Vulvovaginitis due to *Cryptococcus magnus* in Iran

**DOI:** 10.18502/cmm.4.1.32

**Published:** 2018-03

**Authors:** Ali Ghajari, Ensieh Lotfali, Maryam Norouzi, Zahra Arab-Mazar

**Affiliations:** 1 Department of Medical Parasitology and Mycology, School of Medicine, Shahid Beheshti University of Medical Sciences, Tehran, Iran; 2 Infectious Diseases and Tropical Medicine Research Center, Shahid Beheshti University of Medial Sciences, Tehran, Iran

**Keywords:** *Cryptococcus magnus*, Isolation, Ribosomal 28S DNA sequence

## Abstract

**Background and Purpose:**

*Cryptococcus. magnus *is a non-*Cryptococcus neoformans* species isolated from certain plants, soil, air, and arctic glaciers.

**Case report:**

This report describes a case of a 23-year-old Iranian female with pruritus and vaginal irritation. Conventional tests and molecular analysis of the samples of vaginal discharge were performed. The mentioned analyses revealed *Cryptococcus magnus* as the causative agent of vaginal infection. The minimum inhibitory concentration analysis revealed that this species is susceptible to itraconazole, fluconazole, ketoconazole, and amphotericin B. The patient received 200 mg of oral ketoconazole once daily for 10 days. The patient did not show any clinical signs of vaginal infection after six months.

**Conclusion:**

*C. magnus* was found to have the ability to cause vulvovaginitis**.** This is the first report of successful detection and treatment of vulvovaginal infection with *C. magnus*.

## Introduction

Cryptococcosis is characterized by a chronic course and is usually caused by the ubiquitous yeast *Cryptococcus neoformans*, predominantly by its variety *C. neoformans* var. *grubii*, and in endemic areas by *Cryptococcus gattii*. Other *Cryptococcus* species are rarely reported to cause infections and most of them are known to be low or non-pathogenic [1].

In the present case, *Cryptococcus magnus* was isolated from a 23-year-old female patient suffering from a vulvovaginal disease, and the outcome of this uncommon yeast was examined. 

*C. magnus* can clearly be distinguished from other *Cryptococcus* spp. by ribosomal DNA sequence analysis [2, 3]. *C. magnus *has been isolated from certain plants, and it is detectable in soil, air, and even on arctic glaciers. It has also been detected in goat milk, murine intestinal tract, feline ear canal, and human respiratory tract [4]. In most of these reports, the identification of *C. magnus* was confirmed by molecular methods.

Herein, we present a case of vulvovaginitis due to *C. magnus* in a 23-year-old Iranian woman and discuss the biology of this agent. To the best of our knowledge, this is the first report on *C. magnus* vaginal infection.

## Case report

***Case history***

A 23-year-old married female patient presented to one of the hospitals of Damavand city, Tehran, Iran, due to severe itching and vaginal irritation. Vaginal examination revealed thick, curdle-like, white-colored discharge, edema, and intense pruritus of the vulva. The vagina and labia were erythematous.  She was using an intra-uterine device (IUD) as a contraceptive method.

***Diagnosis***

**Figure 1:**
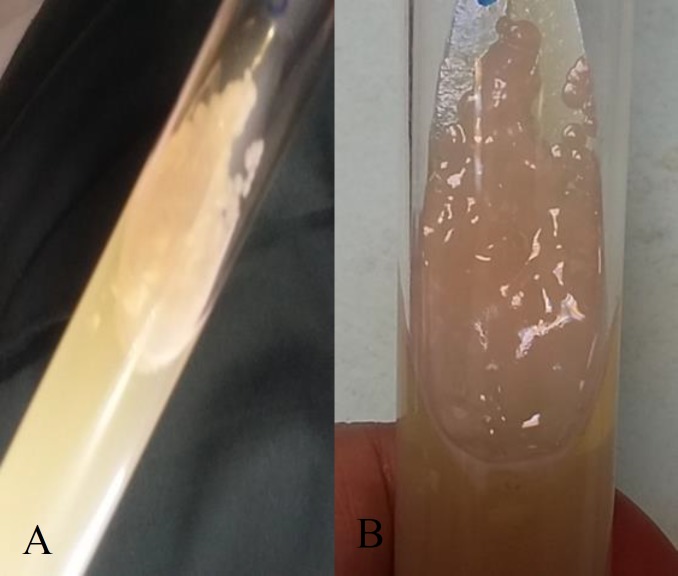
Cream-colored colonies, slightly mucoid (A), and pink-colored colonies after three days on Sabouraud Dextrose Agar (B)

Due to the suspected diagnosis of vulvovaginal candidiasis, fresh samples of vaginal discharge were sent for mycological examination to the Division of Mycology, School of Medicine, Shahid Beheshti University of Tehran, Iran. Vaginal discharge was sampled by using a speculum and sterile swab. The swab was transported to the laboratory into normal saline. Two specimens were obtained under sterile conditions, one for microscopic examination and the other for fungal culture. A slide was prepared for Methylene blue staining. The vaginal swab was inoculated on Sabouraud Dextrose Agar (SDA; Merck, Germany) [5] and incubated at 30°C for 24 h. The produced cream-colored colonies were slightly mucoid, smooth, highly glossy, and slim in texture that were indistinguishable from *Candida* spp. colony. After three days, the cream color changed to pink (Figure 1).

**Figure 2:**
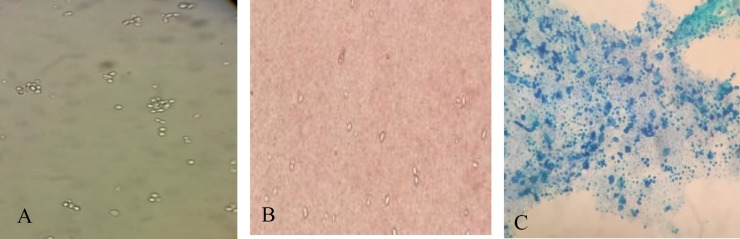
Ovoid to elongate, single or pairs of cells without pseudomycelium (A); stain according to Indian ink (B), Stain according to Methylene blue (C)

Microscopic examination of the culture after 48 h showed round to oval yeast, single or pairs of cells without true hyphae or pseudohyphae and without capsule in Indian ink (Figure 2).

Genomic DNA was extracted from culture using glass bead method [6]. Then, the ITS regions of rDNA gene of isolates was amplified by the universal fungal primers, ITS1 (5´-TCCGTAGGTGAACCTGCGG-3´) and ITS4 (5´-TCCTCCGCTTATTGATATGC-3´) [7]. The PCR product was applied for the accurate identification of isolate (Bioneer, Korea). For confirmation of species identity, the obtained sequences were compared with similar sequences in the open access NCBI database (http://blast.ncbi.nlm.nih.gov/Blast.cgi). Alignment of the obtained sequence in BLAST revealed a 100% identity with the type strain of *Filobasidium magnum*, which is indicated with sequence ID: MG786767.1 and high homology (99%) with *Cryptococcus magnus* with sequence ID: GU237052.1. The sequences were in GenBank under accession number ‘‘MG786767-1.’’

***Antifungal susceptibility***

Antifungal susceptibility tests were performed by broth micro dilution method as described in Clinical and Laboratory Standards Institute [8] guidelines, document M27-S3 [8].* C. parapsilosis *type strain* (*ATCC 22019) was used for quality control in all antifungal susceptibility tests. Tests were performed in 96-well round-bottom microtiter plates. Drug concentration ranges were 0.03 to 64 μg/ml for itraconazole (ITC), fluconazole (FLC), ketoconazole (KTC), and amphotericin B (AMB). Cell suspensions were prepared in RPMI 1640 medium (Invitrogen, USA) and were adjusted to give a final inoculum concentration of about 0.5 × 10^3^ to 2.5 × 10^3^ cfu/ml. The plates were incubated at 35°C for 48 h. Minimum inhibitory concentration (MIC) was then determined and compared with a drug-free control. All the tests were performed in duplicate [8, 9].

The MIC values for ITC, KTC, AMB, and FLC were 0.031 μg/ml, 0.031 μg/ml, 0.062 μg/ml, and 0.062 μg/ml respectively, revealing the sensitivity of the mentioned causative agent. The patient received treatment with topical ketoconazole ointment, but there were no signs of recovery after seven days. Then, the patient was finally treated successfully using 200 mg daily of oral ketoconazole in 10 days. No further clinical signs of vaginal infection were observed after six months. Relapse of the infection was not revealed in two, four, and six-months follow up of the patient. No clinical manifestations were shown at the involved site.

The Ethics Committee of Shahid Beheshti University of Medical Sciences in Iran approved this study (ethics committee code: IR.SBMU.MSPPP.REC.1395.3).

## Discussion

The present report describes an uncommon case of vulvovaginitis caused by vaginal infection with *C. magnus*. This species can be isolated from soil, air, and plants [4, 10]. 

Until now, there has been one published report on the isolation of *C. magnus* from pediatric cancer patients and two published cases from cats [1, 11, 12]. 

Khan et al. reported the isolation of *C. magnus* from nasal specimens of acute lymphoblastic leukemia patients. This species was resistant to caspofungin, anidulafungin, 5-flucytosine, and itraconazole, but it was susceptible to amphotericin B, posaconazole, and voriconazole by E-test [11].

Kano et al. [12] from Japan isolated this yeast from the ear canal of a clinically healthy cat that had a history of otitis externa. *C. magnus* was attributed to *Aspergillus fumigatus*, and then it was successfully cured through antifungal treatment for 25 days. 

In contrast to the report by Kano et al., Poth et al. described a severe case of *C. magnus* infection in an immunocompetent cat. The left foreleg was completely amputated and the cervical lymph node was excised in this male domestic cat due to suspicion of a tumor. Based on histopathological and mycological investigations, response to treatment with 10 mg/kg of fluconazole for five weeks was satisfactory [1].

Infections due to* C. neoformans and C. gattii *have frequently been reported in cats, dogs, and humans, whereas no information is available regarding the course of disease caused by* C. magnus *[13-15].

Generally, the identification of yeasts is limited in histological preparation.* Candida *spp. often show budding yeasts, pseudohyphae, and true hyphae in tissue. *C. neoformans *can be identified based on its capsule [1]. *C. magnus *may look like* Histoplasma capsulatum* in form and size by using routine microscopic techniques. Thus, a reliable method is required for the final identification of yeasts followed by mycological studies and confirmation by PCR and sequencing, especially for unusual pathogens. 

To the best of our knowledge, the case described here is the first report of vulvovaginitis due to *C. magnus*. In the present case, after MIC approval, treatment was initiated with topical ketoconazole ointment, but there were no signs of recovery after seven days. Therefore, 200 mg of oral ketoconazole was administered daily for 10 days, which resulted in successful treatment. Relapse of the infection was not revealed in two-, four-, and six-month follow up of the patient. At the involved site, no clinical manifestations were observed. Although *Cryptococcus *spp. rarely cause vulvovaginitis, there have been some articles on this issue (Table 1).

**Table 1:** Overview of six reported articles of vulvovaginitis due to* Cryptococcus* species (1985- 2018)

**NO**	**Age/year**	**Location**	**No. Total/** **case samples**	**Agent**	**Clinical presentation**	**Examination**	**Treatment**	**Case characteristics**	**Reference**
1	NI/ 1985	USA	805/1	*Cryptococcus. ungulaticus*	Vulvovaginal complaints	Culture	NI	NI	[16]
2	60/1987	USA	Case report	*Cryptococcus. neoformans*	Cutaneous lesion	Culture and Biopsy	NI	Renal transplant recipient	[17]
3	72/1993	NI	Case report	*Cryptococcus. spp*	Colon cancer	Culture	FLC	Colon cancer	[18]
4	20/2005	Malaysia	Case report	*Cryptococcus. neoformans*	Vulvovaginal complaints	Biopsy	Oral FLC	NI	[19]
5	NI/2009	Saudi Arabia	1000/1	*Cryptococcus. neoformans*	NI	Culture and API20C kit	NI	NI	[20]
6	23/2018	Iran	Case report	*Cryptococcus. magnus*	severe itching and vaginal irritation	Culture and sequencing	KCZ	immunocompetent	Current case

NI =Not indicated; spp,*, *species; FLU, fluconazole; ketoconazole, KCZ

## Conclusion

*C. magnus* was found to have the ability to cause vulvovaginitis. This report could be of clinical significance and helpful for the management of similar cases. The case presented here is the first report of successful detection and treatment of vulvovaginal infection with *C. magnus*. 
